# P05 Identifying novel drug targets of the ESKAPE pathogen *Pseudomonas aeruginosa*

**DOI:** 10.1093/jacamr/dlad143.009

**Published:** 2024-01-03

**Authors:** Ben Scott, Barrie Wilkinson

**Affiliations:** John Innes Centre; John Innes Centre; Isomerase Therapeutics

## Abstract

The Gram-negative ESKAPE pathogen *Pseudomonas aeruginosa* is currently listed top of the priority one pathogens by the WHO that require urgent research and development of new antimicrobials. Currently, microbiologists and clinicians face an ever-growing challenge in treating MDR bacterial infections, for this, and other pathogens, using conventional antibiotics. With a limited number of antibiotics currently in clinical trials and a general stagnation in the discovery of promising antimicrobials, new drug targets are desperately needed, and recent investigations include targeting bacterial virulence factors. Targeting bacterial virulence factors should reduce the pathogenesis of an organism, slow its infection progression and prevent vital virulence processes such as biofilm formation as well as potentially increasing a strain’s susceptibility to antibiotics. However, given the incredible diversity of bacteria, virulence mechanisms vary massively, meaning that identifying a broad-spectrum virulence target presents a major challenge. This important point has brought researchers to a fascinating group of enzymes that are ubiquitous in nature, and possess a plethora of biological roles, including acting as virulence factors. The macrophage infectivity potentiators (Mips) belong to the ubiquitous FK506 binding protein (FKBPs) family of peptidyl-prolyl cis/trans isomerases (PPIases). FKBPs are found in all bacteria serving as general house-keeping proteins involved in protein folding and chaperoning. However, a small subset has been identified as virulence proteins. Mips have been characterized in several Gram-negative pathogens including *Legionella pneumophila* and *Burkholderia pseudomallei* and were shown to be essential for invasion of macrophages, both *in vitro* and in murine-infection models, and for other virulence determinants. *Pseudomonas aeruginosa* also possess three Mip virulence factors, PaMip1, PaMip2 and PaMip3, which are required for the full virulence of *P. aeruginosa* in *in vivo* and *ex vivo* models. Virulence can be addressed in a pharmacological manner using bacterial natural products.

